# Divergent Roles of Amino Acid Residues Inside and Outside the BB Loop Affect Human Toll-Like Receptor (TLR)2/2, TLR2/1 and TLR2/6 Responsiveness

**DOI:** 10.1371/journal.pone.0061508

**Published:** 2013-04-23

**Authors:** Yuan Qiu, Yan Ding, Lingyun Zou, Zhangping Tan, Taiping Liu, Xiaolan Fu, Wenyue Xu

**Affiliations:** 1 Department of Pathogenic Biology, Third Military Medical University, Chongqing, P. R. China; 2 Department of Microbiology, Third Military Medical University, Chongqing, P. R. China; 3 Institute of Immunology, PLA, Third Military Medical University, Chongqing, P. R. China; Centre d'Immunologie de Marseille-Luminy, CNRS-Inserm, France

## Abstract

TLR2 specifically recognizes a wide range of ligands by homodimerizing or heterodimerizing with TLR1 or TLR6. However, the molecular basis of the specific signalling transduction induced by TLR2 homodimerization or heterodimerization with TLR1 or TLR6 is largely unknown. In this study, we found three amino acid residues, two (663L and 688N) outside and one (681P) inside the BB loop, which were conserved in all of the TLRs, except for the TLR3 toll/IL-1R(TIR) domain. The responsiveness of human TLR2/2, TLR2/1 or TLR2/6 was completely lost when 663L and 688N were replaced with the corresponding amino acid residues in the TLR3 TIR domain, respectively. However, the response of TLR2 (P681A) to the high concentration of TLR2/TLR6 agonist was almost intact, but the activity of TLR2 (P681A) was greatly reduced when stimulated with the TLR2/1 agonist or the TLR2/2 agonist. Although the surface expression of TLR2 (L663E) was sharply reduced, both the intracellular distribution and the surface expression of all of the other TLR2 mutants were unchanged. The ability of all three TLR2 mutants to recruit MyD88, was consistent with their responsivenesses. Computer modelling indicated that the surface negative charge of all of the TLR2 mutants' BB loops was reduced. Thus, our data demonstrated that the 663L and 688N residues outside of the BB loop were essential for the responsiveness of TLR2/2, TLR2/1 and TLR2/6, but the 681P residue inside of the BB loop exhibited divergent roles in TLR2/2, TLR2/1 and TLR2/6 signalling transduction, thereby providing clues regarding the specific signalling transduction of TLR2/2, TLR2/1 and TLR2/6.

## Introduction

TLRs (Toll-like receptors) are the main pattern recognition receptors (PRRs) on myeloid cells, and they have an important role in detecting diverse invading microorganisms and eliciting early innate immune responses[1]. There have been 10 functional TLRs identified in humans, and most of these TLRs can specifically recognize different pathogen-associated molecular patterns (PAMPs). Of the TLRs, TLR2 is involved in recognizing a wide range of ligands, including peptidoglycan and lipoteichoic acid from gram-positive bacteria [2], lipoarabinomannan from mycobacteria [3], zymosan from fungi [4] and GPI from *plasmodium falciparum* [5].

TLR2 can also discriminate triacylated lipopeptides from diacylated lipopeptides through heterodimerization with TLR1 or TLR6. For example, the TLR2-TLR1 heterodimer recognizes triacylated lipopeptides from gram-negative bacteria and mycoplasma [6], whereas the TLR2-TLR6 heterodimer recognizes diacylated lipopeptides from gram-positive bacteria and mycoplasma [7]. Although the molecular basis of the specific ligands recognized by TLR1/2 and TLR2/6 has recently been revealed by crystal structure analysis [8], [9], there is little known about the mechanism of signalling transduction specifically mediated by TLR2/1, TLR2/2 and TLR2/6.

TLR is a type I transmembrane protein. The extracellular domain of TLR contains leucine-rich repeats that act as scaffolds for ligand recognition. The intracellular domain that shares a highly homologous structure with that of IL-1R is referred as the toll/IL-1R (TIR) domain [10]. It is well-known that the recruitment of the downstream adaptor, Myeloid differentiation primary response gene 88 (MyD88), to the TIR domain is a critical step for the signalling transduction of TLR2 [11]. Analysis of both crystal structures and functional assays suggest an essential role for the BB loop in the interaction between the TLR2 TIR domain and its downstream adaptors [12]. In addition to the BB loop, amino acid residues outside of the BB loop, such as the Pococurante (poc) site (V660N) [13], were also reported to be critical for downstream adaptor recruitment and TLR2 signalling transduction[14]. However, a previous study indicates that the BB loop in the TLR2 TIR domain is intrinsically flexible [15], which implies that the binding of different ligands to the ectodomain of TLR2 might cause different conformational changes and may lead to distinct interactions with the adaptor, MyD88. The different response of TLR2/2, TLR2/1 and TLR2/6 ligand, in the context of the Poc mutant further supports for the proposition of BB loop conformational changes and distinct interactions with MyD88 [13]. Therefore, we aimed to identify the amino acid residues outside or inside the BB loop that might be involved in the recruitment of the downstream adaptors and signalling transduction of TLR2/1, TLR2/2 or TLR2/6.

In this study, we identified one amino acid residue inside the BB loop and two other residues in the bilateral region of the BB loop that were conserved in all of the TLRs but not in the TLR3 TIR domain, and we investigated their role in the responsiveness of human TLR2/2, TLR1/2 or TLR2/6. An NF-κB gene reporter activity assay showed that two amino acid residues outside of the BB loop were essential for the responsiveness of human TLR2/2, TLR1/2 and TLR2/6. However, one amino acid residue inside the BB loop exhibited divergent roles in the responsiveness of human TLR2 heterodimerization with TLR1 or TLR6 when the site was replaced with an amino acid residue corresponding to the TLR3 TIR domain. The expression level, intracellular distribution, ability to recruit MyD88 and computer modelling of all of the mutants were also assessed.

## Materials and Methods

### Ethics statement

Ethics approval for the collection of human peripheral blood in this study was obtained by ethical committee of Third Military Medical University. Healthy donors were recruited on their own will, and wrriten consent was obtained from each participant.

### Plasmids construction and Site-Directed Mutagenesis

The full-length human TLR2 coding sequence was amplified from human PBMCs and was cloned into the *Hind* III/*BamH* I site of pFLAG-CMV8 (Sigma). Full-length MyD88 was amplified from HEK293T cells (ATCC) and then cloned into the *Hind*III/*Xba*I site of pCMV-HA (Beyotime). Mutants of TLR2 (N657E), TLR2 (L663E), TLR2 (P681A) and TLR2 (N688A) were constructed using a Site-Directed Mutagenesis Kit (Qiagen) according to the manufacturer's instructions. All of the constructs and mutants were verified by sequencing.

### Cell culture and flow cytometry assay

Human embryonic kidney 293T (HEK293T) cells were cultured in Dulbecco's modified Eagle's medium (DMEM) supplemented with 10% heat-inactivated FBS, 100 U/ml penicillin and 100 µg/ml streptomycin at 37°C under humidified air containing 5% CO_2_.

Next, 1×10^5^ HEK 293T cells/well were plated in a 24-well plate. Each well received 0.4 µg of either human wild-type TLR2, TLR2 (N657E), TLR2 (L663E), TLR2 (P681A) or TLR2 (N688A) and was transfected with Lipofectamine 2000 (Invitrogen). At 24 h post-transfection, the cells expressing human wild-type TLR2 and its mutants were incubated with Biotin anti-human TLR2, clone TL2.1 (eBioscience) or isotype Biotin IgG2a (eBioscience), stained with streptavidin-PE (eBioscience) and analysed by FACS.

### Dual-luciferase assay for NF-κB

For this assay, 1×10^5^ HEK 293T cells/well were plated in a 24-well plate. Each well received 0.2 µg of either human wild-type TLR2, TLR2 (N657E), TLR2 (L663E), TLR2 (P681A) or TLR2 (N688A) along with 2 ng of TK-RL (Promega) and 200 ng of the reporter gene, pBIIx-luc (a gift from Dr Sankar Ghosh's Lab, Yale University) and were transfected with Lipofectamine 2000 (Invitrogen). At 24 h post-transfection, the cells were stimulated with an increased concentration of the TLR2/2 agonist, LTA (Invivogen), the TLR2/1 agonist, Pam3CSK4 (Invivogen) or the TLR2/6 agonist, FSL (Invivogen) for 6 h, respectively. Then cells were lysed, and both firefly and Renilla luciferase activities were determined using a Dual Luciferase Assay kit (Promega).

### Confocal immunofluorescence assay

HEK293T cells were seeded on a cover slide and were transfected with either human wild-type TLR2, TLR2 (N657E), TLR2 (L663E), TLR2 (P681A) or TLR2(N688A). At 24 h post-transfection, the cells were incubated with FITC-labelled anti-FLAG (Sigma) and DAPI (Beyotime) and observed by confocal fluorescence microscopy.

### Immune-precipitation and western blotting

HEK293T cells were co-transfected with either human wild-type TLR2, TLR2 (N657E), TLR2 (L663E), TLR2 (P681A) or TLR2 (N688A) and MyD88-HA. At 24 h post-transfection, the cells were stimulated with 100 ng/ml of LTA, 100 ng/ml of Pam3CSK4 or 1 µg/ml of FSL for 30 min, respectively. The cells were then lysed in lysis buffer [containing 50 mM Tris-HCl, pH 7.5, 100 mM NaCl, 0.1% Triton X-100, 1 mM 1, 4-dithiothreitol and a proteinase inhibitor mixture (Sigma)] and centrifuged at 4°C. The supernatant was collected, and 500 µg of protein was subsequently incubated with anti-FLAG-conjugated beads (Sigma) for 2 h at 4°C. The bound protein was washed five times in lysis buffer. The proteins were eluted by boiling the samples in SDS sample buffer, and then separated by SDS-PAGE on 10% gels. After the transfer, polyvinylidene difluoride (PVDF) filters were immunoblotted using anti-HA (Signalway antibody) and anti-FLAG polyantibody (Sigma) and was developed with enhanced chemiluminescence (ELC) (Perice).

### Molecular modelling

All human TLR2 mutants were homology modelled according to the known crystal structure of the TLR2 TIR domain [12]. Molecular dynamics simulation of all of the human TLR2 mutants was performed using the NAMD2.9 and VMD1.9.1 software programmes. The software programme Pymol was used to construct the molecular surface charges for all of the human TLR2 mutants.

### Statistical analysis

Differences between wild-type human TLR2 and the TLR2 mutants were analysed using the SPSS 12.0 software. For the unpaired Student's t-test, P values<0.05 were regarded as statistically significant.

## Results

### Three amino acid residues conserved in all TLRs but not TLR3 TIR domain

**Figure 1:**
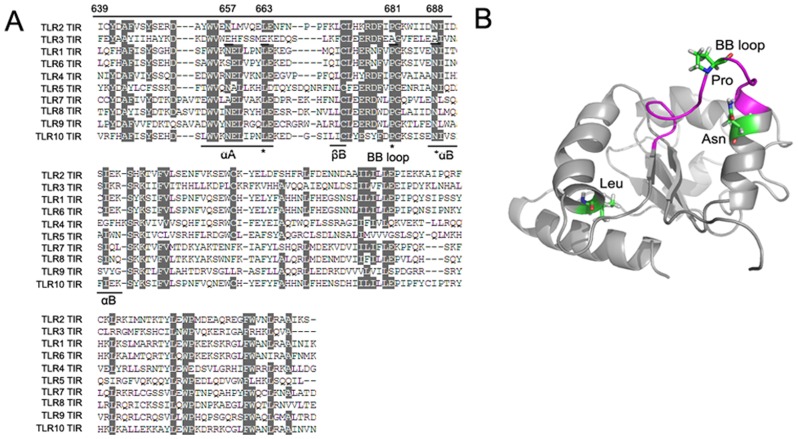
Conserved amino acid residues in all but not human TLR3 TIR domain and their location in the crystal structure of human TLR2 TIR. A, All of the TIR domains, including human TLR2, TLR3, TLR1, TLR6, TLR4, TLR5, TLR7, TLR8,TLR9 and TLR10, were aligned. The sequences responsible for the structure of αA, βA, αB, and the amino acid residues specific for human TLR3 TIR and one (657 E) specific for TLR3, but semi-conserved in all other human TLRs TIRs were underlined. The BB loop was indicated as the sequence between βA and αB, and three conserved amino acid residues in all of the TLRs, except for the TLR3 TIR domain, such as L, P and N, were indicated as“*”. The numbers indicate the positions of the amino acid residues in human TLR2. B, Stick representation of all three conserved amino acid Leu (663), Pro (681) and Asn (688), and highlighting BB loop (*pink*) in the cystal structure of human TLR2 TIR domain.

All of the TLRs (TLR1, TLR2, TLR4, TLR5, TLR6, TLR7/8,TLR9 and TLR10), except for TLR3, share the MyD88-dependent pathway through the recruitment of MyD88 by their TIR domains. However, the TLR3 TIR domain can only recruit TIR-domain-containing adapter-inducing interferon-β (TRIF), leading to the activation of the MyD88-independent pathway. Thus, it is the TIR domain that determines the selective activation of the MyD88-dependent and/or -independent pathway by TLRs through recruiting different adaptors [1]. We proposed that amino acid residues conserved in all of the TLRs but not in the TLR3 TIR domain might be essential for MyD88-dependent signalling transduction. TIR domain alignment of human TLR2 with the other TLRs revealed three amino acid residues, 663L, 681P and 688N, which were conserved in all of the TLRs, except the TLR3 TIR domain. According to the structure of the TLR2 TIR domain [12], residue 681P was located in the middle of the BB loop, which is regarded as the TIR interaction surface between IL-1/TLRs and MyD88 [11]. However, both 663L and 688N were found outside the BB loop and were located in the αA helix and αB sheet of the TIR domain, respectively (Fig. 1).

### Essential role of two amino acid residues outside the BB loop in the responsiveness of human TLR2/2, TLR2/1 and TLR2/6

**Figure 2:**
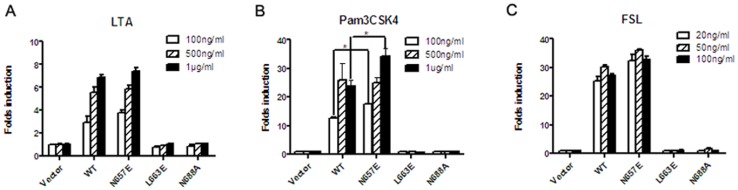
Responsiveness of human TLR2 (N657E), TLR2 (L663E) and TLR2 (N688A) to TLR2/2, TLR2/1 and TLR2/6 agonists. HEK293T cells in 24-well plates were transfected with human TLR2 (N657E), TLR2 (L663E) or TLR2 (N688A), and TK-RL and pBIIx-luc. At 24 h post-transfection, the cells were stimulated with the indicated concentrations of LTA (A), Pam3CSK4 (B) and FSL (C) for 6 h, respectively, and then both firefly and Renilla luciferase activities were determined using a dual-luciferase assay. The experiments were repeated for three times, and all of the data were expressed as the mean ± SD. * indicated as *p*<0.05.

To assess the role of the above conserved amino acid residues in the responsiveness of human TLR2, we replaced 663L, 681P and 688N with amino acid residues glutamic acide (E), alanine (A) and alanine (A), which were the corresponding residues in the TLR3 TIR domain, respectively. Additionally, a semi-conserved amino acid residue asparagine (N) at position 657 was selected as a control and replaced with glutamic acid (E). Because HEK293T cells express both TLR1 and TLR6 endogenously [16], wild-type TLR2 or each of TLR2 mutants was only transfected to test the responsiveness of TLR2/1, TLR2/2 or TLR2/6 in the following experiments. As a result, the NF-κB gene reporter activity of human TLR2 (N657E) was significantly increased compared to that of the wild-type TLR2 following stimulation with the TLR2/1 agonist, Pam3CSK4 (Fig. 2B, *p<0.05*), but shows similar response with that of wild-type TLR2 following sitmulation with either the TLR2/2 agonist, LTA (Fig. 2A), or the TLR2/6 agonist, FSL (Fig. 2C). However, both TLR2 (L663E) and TLR2 (N688A) completely lost their responsiveness to increasing concentrations of LTA (100 ng/ml, 500 ng/ml and 1 µg/ml), Pam3CSK4 (100 ng/ml, 500 ng/ml and 1 µg/ml) or FSL (20 ng/l, 50 ng/ml and 100 ng/ml) (Fig. 2). Thus, our data suggested that the conserved amino acid residues 663L and 688N were essential not only for human TLR2/2 but also for TLR2/1 and TLR2/6 signalling transduction.

### Differential effects of 681P inside the BB loop on the responsiveness of human TLR2/2, TLR2/1 and TLR2/6

**Figure 3:**
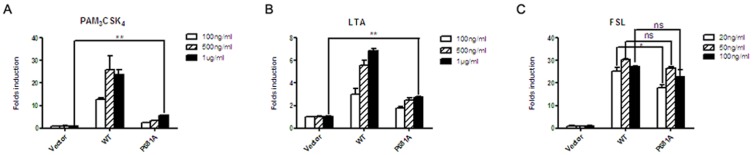
Responsiveness of human TLR2 (P681A) to TLR2/2, TLR2/1 and TLR2/6 agonists. HEK293T cells in 24-well plates were transfected with TLR2 (P681A), TK-RL and pBIIx-luc. At 24 h post-transfection, the cells were stimulated with the indicated concentrations of LTA (A), Pam3CSK4 (B) and FSL (C) for 6 h, respectively, and then both firefly and Renilla luciferase activities were determined using a dual-luciferase assay. The experiments were repeated for three times, and all of the data were expressed as the mean ± SD. *P<0.05, ** *P*<0.01, ns, not significant.

It is well-known that TLR2 can discriminate triacylated lipopeptides from diacylated lipopeptides through heterodimerization with TLR1 or TLR6 [8], [9]. However, little is known about how the signalling is specifically transduced by TLR2/1 and TLR2/6. Because neither residue 663L nor 688N could divergently influence the signalling transduction of TLR2/2, TLR2/1 and TLR2/6, we subsequently determined the effect of the 681P residue on the responsiveness of TLR2/2, TLR2/1 and TLR2/6, respectively. As shown in Fig. 3, the response of TLR2 (P681A) to the TLR2/2 agonist, LTA and the TLR2/1 agonist, Pam3CSK4 were largely suppressed at all of the tested concentrations. However, its response to high concentrations of the TLR2/6 agonist, FSL (50 ng/ml and 100 ng/ml) was equivalent to that of wild-type TLR2, although a significant suppression in responsiveness was observed following stimulation with 20 ng/ml of FSL (*p<0.05*). Thus, these data supported the divergent role of 681P in the responsiveness of TLR2/2, TLR2/1 and TLR2/TLR6.

### Except for TLR2(L663E), both the expression level and the intracellular distribution of all of the human TLR2 mutants were unaltered

**Figure 4:**
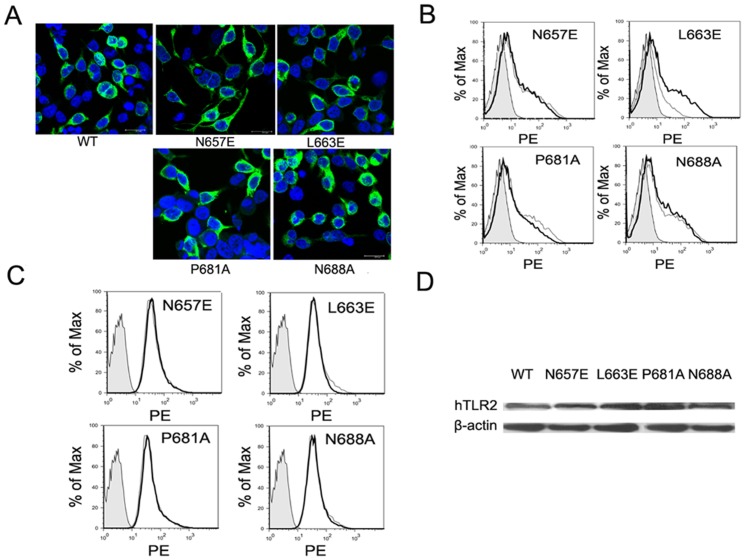
Expression and intracellular distribution of the human TLR2 mutants. HEK293T cells in 24-well plates were transfected with each indicated mutant and analysed 24 h later. A, the cells were stained with FITC-labelled anti-FLAG and DAPI and observed under confocal fluorescence microscopy. B, the surface expression of wild-type TLR2 (*solid line*) and each of the TLR2 mutants (*thin line*) on HEK293 was detected by FACS. C, the expression of wild-type TLR2 (*solid line*) and each of the TLR2 mutants (*thin line*) was detected following permeabilisation of the cell membrane. D, the expression of each of the TLR2 mutants was determined by western blot, with β-actin as an internal control. Each experiment was repeated for three times, and one was represented.

A previous study reported that the hyporesponsiveness of a TLR4 mutant may result from its reduced expression or from the alteration of its intracellular distribution [17]. Thus, both the expression and intracellular distribution of all of the mutants were investigated. All of the human TLR2 mutants, including TLR2 (N657E), TLR2 (L663E), TLR2 (P681A) and TLR2 (N688A), were mainly expressed at the cell membrane and the cytoplasm (Fig. 4A), which is the same distribution as wild-type TLR2. The expression levels of both TLR2 (P681A) and TLR2 (N688A) on the surface of the HEK293T cells were at comparable levels to the wild-type TLR2 expression, However, the expression of TLR2 (L663E) on the surface of the HEK293T cells was greatly reduced (Fig. 4B), which might be associated with its hyporesponsiveness to all three of the TLR2 agonists. In contrast to their surface expressions, the total levels of all of the human TLR2 mutants in HEK293T cells were similar to that of the wild-type TLR2 expression, as demonstrated flow cytometry following permeabilization of the cell membranes, or much higher than that of the wild-type TLR2 expression indicated by Western blot (Fig. 4C,D).

### The ability of all of the human TLR2 mutants to recruit the downstream adaptor, MyD88

**Figure 5:**
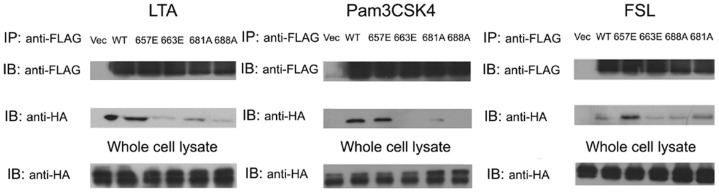
The ability of all of the TLR2 mutants to recruit MyD88. HEK293T cells were co-transfected with empty vector pFLAG-CMV8, FLAG-tagged wild-type TLR2 or each of the TLR2 mutants, along with MyD88-HA. At 24 h post-transfection, the cells were stimulated with LTA, Pam3CSK4 or FSL for 30 min. The cells were lysed, and the extracts were immunoprecipitated by anti-FLAG conjugated beads. TLR2 and MyD88 were subsequently detected in the immunoprecipitated proteins by western blot with an anti-FLAG antibody and an anti-HA polyclonal antibody. The quantity of MyD88 in the whole cell lysates was also detected with the anti-HA polyclonal antibody. The experiment was repeated for three times, and one was represented.

Next, we tested whether the different responses of the TLR2 mutants to the TLR2/2, TLR2/1 and TLR2/6 agonists resulted from their abilities to recruit the downstream adaptor, MyD88. HEK293T cells were co-transfected with MyD88-HA and FLAG-tagged wild-type TLR2 or each TLR2 mutant, and the recruitment of MyD88 was determined by immunoprecipitation following Pam3CSK4, FSL or LTA stimulation, respectively. As shown in Fig. 5, TLR2 (N657E) retained its capacity to recruit MyD88 following stimulation with either TLR2/2, TLR2/1 or TLR2/6 agonists. In contrast to TLR2 (N657E), the abilities of both TLR2 (L663E) and TLR2 (N688A) to recruit MyD88 was greatly suppressed following stimulation with LTA, Pam3CSK4 or FSL. However, in the case of TLR2 (P681A), the mutant's ability to recruit MyD88 was partially suppressed when stimulated either with LTA or Pam3CSK4, and its recruitment capabilities remained almost intact when stimulated with FSL. These data were highly consistent with the results of the responsiveness of the human TLR2 mutants, suggesting that the responses of the TLR2 mutants were strongly linked to their differing capacities for recruiting their downstream adaptors.

### Mutation changes the surface charge and conformation of the BB loop of the TLR2 TIR DOMAIN

**Figure 6:**
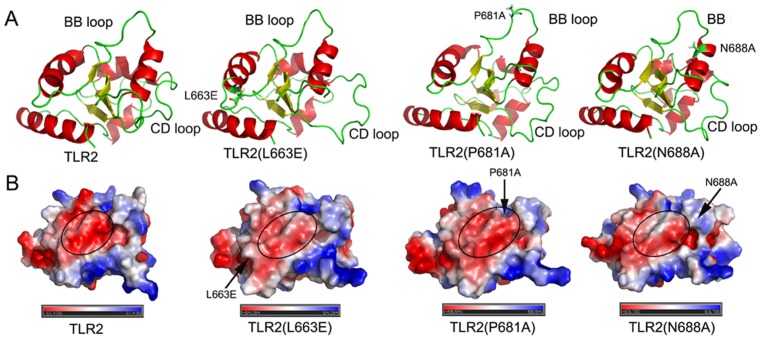
Electrostatic charge and conformation of the BB and CD loop were compared between wild-type TLR2 and its mutants. A, the conformation of the BB and CD loop in wild-type TLR2, TLR2 (L663E), TLR2 (P681A) and TLR2 (N688A) was compared, and the mutated residues L663E, P681A and N688A were stick represented. B, comparison of the electrostatic surfaces of wild-type TLR2, TLR2 (L663E), TLR2 (P681A) and TLR2 (N688A), the change of positive charge (*blue*) and negative charge (*red*) in the BB loop was indicated by a circle, and the mutated residues L663E, P681A and N688A were indicated by an arrow.

To understand why mutating the above three amino acid residues greatly influenced the capacity of TLR2 to recruit MyD88 and its responsiveness, computer modelling of wild-type TLR2 and all of the TLR2 mutants was performed using the known crystal structure of TLR2 [12]. As shown in Fig. 6A, mutating the 663L residue changed the shape of the CD loop, but not the BB loop, of the TLR2 TIR domain. However, mutating either 681P or 688N altered the shape of the BB loop in the TLR2 TIR domain. In the case of the electrostatic charge of the BB loop surface, either TLR2 (L663E), TLR2 (P681A) or TLR2 (N688A) lost negative charge to various degrees compared to that of wild-type TLR2 (Fig. 6B). Because the BB loop is critical for the interaction of the TLR2 TIR domain with MyD88 [10], our data suggested that the conserved amino acid residues outside of the BB loop might interfere with the TLR2 signalling transduction indirectly by altering the electrostatic charge and/or the conformation of the BB loop.

## Discussion

TLR2 is involved in the specific recognition of a wide range of ligands through homodimerization or heterodimerization with TLR1 or TLR6. Although crystal structure analysis has revealed the basis by which TLR2/1 and TLR2/6 recognize the specific ligands [8], [9], the mechanism of the specific signalling transduction is still poorly understood. In the present study, we reported that two amino acid residues outside of the BB loop of the TIR domain were essential for the signalling transduction of TLR2/2, TLR2/1 and TLR2/6. However, in contrast to the two amino acid residues outside of the BB loop, the amino acid residue 681P inside of the BB loop had diverse effects on the responsiveness of TLR2/2, TLR2/1 and TLR2/6 when it was replaced with alanine (A). Further analysis suggested that the different influences of 663L, 681P and 688N on the responsiveness of TLR2/2, TLR2/1 and TLR2/6 were associated with the alteration of the BB loop conformation and the ability to recruit MyD88.

Recruitment of the downstream adaptor, MyD88, to the TLR2 TIR domain is critical for its signalling transduction [10]. Crystal structure analysis suggested that the essential role of the BB loop in the TIR domain is in the interaction of TLRs with MyD88 [12], and a TLR2-specific cell-permeable BB loop peptide was found to block the signalling transduction of TLR2 following stimulation with Pam2CSK4 [18]. However, we found that the replacement of 681P in the BB loop with A did not completely abrogate the responsiveness of TLR2/1 and TLR2/2 (Fig. 3), which was inconsistent with the results of a mutation wherein proline (P) was replaced with Histidine (H) at the same position. A previous study showed that human TLR2 could not recruit MyD88 when the P at position 681 was replaced with H [12], [13]. Because the surface expression level and intracellular distribution of TLR2 P681A were unaltered (Fig. 4), the discrepancy could be explained as we replaced 681P with a hydrophobic amino acid residue A, and 681P was replaced with hydrophilic H in their study. Previous computer modelling of TLR2 indicated that the replacement of an amino acid residue with a residue containing different hydrophilicity or hydrophobicity would differentially affect the surface charge of the BB loop, their binding to the MyD88 TIR domain and TLR2 signalling transduction [15].

Substantial progress has been made to elucidate the mechanism by which TLR1/2 and TLR2/6 discriminate triacylated lipopeptides from diacylated lipopeptides [8], [9]. However, little is known about how these receptors' specific signalling was transduced. A previous study revealed that a poc site V660N at the αA helix of the TLR2 TIR domain was critical for the signalling transduction of TLR1/2 and TLR2/2 but not for TLR2/6 signalling [13]. Interestingly, we also found a divergent role of the 681P residue in the BB-loop in its effects on the signalling transduction between TLR2/2, TLR1/2 and TLR2/6 (Fig. 3), which is in agreement with a previous report showing that a TLR2-BB loop peptide had different effects on TLR2 signalling transduction following homodimerization or heterodimerization with TLR1 or TLR6 [18]. However, the mechanism of the diverse responsiveness of TLR2 (P681A) to TLR2/2, TLR1/2 and TLR2/6 agonists is still unclear. Previous study demonstrated that TLR2, but not TLR1 or TLR6 TIR domain, is responsible for the binding of MyD88 [19]. In addition, computer modelling indicated that the BB loop in the TLR2 TIR domain was flexible [15], implying that the binding of TLR2/2, TLR1/2 or TLR2/6 agonist would lead to different conformational changes in the BB loop. As located at the tip of the BB loop according to the TLR2 TIR crystal structure [12], 681P was might be differentially involved in the interaction of downstream adaptor MyD88 when it heterodimized with TLR1 or TLR6, resulting in divergent responsiveness of TLR2 (P681A) to TLR1/2 and TLR2/6 agonists. In addition, it is interesting for us to investigate the role of other surface-exposed residues of BB loop in the specific transduction of TLR2/6 signaling in the nearly future.

Although mutations of several amino acid residues outside of the BB loop, such as 631H [16], 677R [20], [21], 713C [22], 715Tyr [14] and 753R [23], [24], [25], were reported to be indispensable for TLR2 signalling transduction and associated with tuberculosis, leprosy, staphylococcal infection or atopy risk, none of these residues was demonstrated to influence the interaction of the TLR2 TIR domain with MyD88, either directly or indirectly. An alanine scanning mutagenesis of the TLR2 DD loop also has identified four residues, 748R, 749F, 752L, and 753R. were crucial for TLR2/1 signaling, but they might be involved in the TLR1/TLR2 heterodimerization [26]. A poc site, 660V, which is critical for TLR2 signaling, was the only site supposed to directly interact with MyD88 [13]. In this study, we determined that two additional amino acid residues, 663L and 688N, which were separately located in the αA helix and αB sheet of the TIR domain, were essential not only for the recruitment of MyD88 and the responsiveness of TLR2/2 and TLR2/1 but also for the activity of TLR2/6 (Fig. 2 and Fig. 5). In contrast to the surface exposure of 681P, both 663L and 688N were buried inside the hydrophobic core of TLR2 TIR domain according to the crystal structure 1FYW [12], and mutation of either of those two residues was supposed to disturb the structure stability of TLR2 TIR domain. This was supported by the computational model of TLR2 mutants, which showed that either TLR2 (L663E) or TLR2 (N688A) lost more negative surface charge of the TLR2 BB loop than that of TLR2 (P681A) (Fig. 6). Compared to TLR2 (P681A), the ability of TLR2 (L663E) or TLR2 (N688A) to recruit MyD88 was greatly reduced, indicating that mutation of either 663L or 688N might block the interaction between the TLR2 TIR domain and MyD88 by indirectly affecting the surface charge of the BB loop (Fig. 5). Because mutating 663L also greatly reduced the surface expression of TLR2 (Fig. 4B), this residue's role in the hyporesponsiveness of TLR2 and reduced recruitment of MyD88 could not be excluded. However, the mechanism of the reduced expression of TLR2 (L663E) on the cell surface was not revealed by our study. Because the total expression level of TLR2 (L663E) was comparable to that of wild-type TLR2, we proposed that TLR2 (L663E) may fail to bind a certain protein in the cytoplasm, such as gp96 [27], [28], which is necessary to traffic to the cell membrane.

Collectively, we have successfully identified three amino acid residues that play an important role in the signalling transduction of TLR2/2, TLR2/1 and TLR2/6 through alignment of TLR2 with the other TLRs TIR domains. Initially, we demonstrated that two amino acid residues outside of the BB loop were essential for either TLR2/2, TLR2/1 or TLR2/6 signal transduction, implying the important role of residues outside of the BB loop in MyD88 recruitment by TLR2. Additionally, we provided evidence to support that the amino acid residue 681P in the BB loop could specifically discriminate between the signalling transduction of TLR2/2, TLR1/2 and TLR2/6. Although we have explained the possible mechanism of the effect of amino acid residue mutation on the signalling transduction of TLR2/2, TLR1/2 and TLR2/6 based on the computer modelling and crystallographic data, circular dichroism (CD) analysis [29] is still required to further validate the secondary structure alteration of all TLR2 mutants. This study helps us to understand the specific mechanism of the signalling transduction of TLR2/2, TLR2/1 and TLR2/6, and it may provide us with novel clues to design targeted therapeutics against TLR2/2-, TLR1/2- and TLR2/6-induced sepsis.
