# Expression of Concern: Divergent Roles of Amino Acid Residues Inside and Outside the BB Loop Affect Human Toll-Like Receptor (TLR)2/2, TLR2/1 and TLR2/6 Responsiveness

**DOI:** 10.1371/journal.pone.0306096

**Published:** 2024-06-21

**Authors:** The PLOS ONE Editors

After this article [1] was published, concerns were raised about Fig 4.

Specifically:

- In the B-actin panel in Fig 4D, lanes 1 and 2 appear similar to lanes 4 and 5, and there appears to be a discontinuity in the background between lanes 3 and 4.
- The gray peaks in Fig 4B panels N657E, L663E, P681A and N688A appear similar.
- The gray peaks in Fig 4C panels N657E, L663E, P681A and N688A appear similar.

The corresponding author stated that the original western blots underlying Fig 4D are no longer available but they identified alternative data provided here as S1and S2 Files. While uncropped underlying images are not available for the alternative western blots provided for Fig 4D (S1 File), quantitative analysis for these alternative blots (S2 File) suggest that the published results are supported. In the absence of the original data underlying Fig 4D, these concerns are not fully resolved.

Additionally, the corresponding author stated that the similarities seen in the gray peaks in all panels in Figs 4B and 4C are due to the use of the same isotype-matched control for each mutant cell sample. The corresponding author stated that the original flow cytometry data for Figs 4B and 4C are no longer available. A member of the *PLOS ONE* Editorial Board assessed this issue and advised that it is not best practice to use the same isotype control for differentially transfected cell lines. They indicated that the results would most likely remain supported, although concerns remain in the absence of a separate isotype-matched control for each sample.

The corresponding author stated that the raw data underlying all results reported in the article are no longer available.

The *PLOS ONE* Editors issue this Expression of Concern to notify readers of the above concerns and relay the supporting data and alternative figure provided by the corresponding author.

## Supporting information

S1 File Alternative western blot for Fig 4D. This file shows the alternative western blot provided for Fig 4D showing expression of each of the TLR2 mutants, with β-actin as an internal control.
(JPG)

S2 File Underlying quantitative data for alternative western blot presented in S1 File. This file contains densitometry analysis for the single western blot experiment presented in S1 File, showing expression of each of the TLR2 mutants, with β-actin as an internal control.
(XLSX)
