# Supplementary figures and images for: Expression of Concern: Divergent Roles of Amino Acid Residues Inside and Outside the BB Loop Affect Human Toll-Like Receptor (TLR)2/2, TLR2/1 and TLR2/6 Responsiveness
Source: PLoS One. 2024 Jun 21;19(6):e0306096. doi: 10.1371/journal.pone.0306096 (PMC11192330; doi:10.1371/journal.pone.0306096)

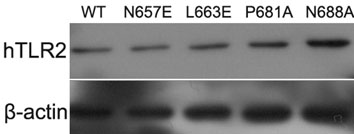

Supplement: S1 File — This file shows the alternative western blot provided for Fig 4D showing expression of each of the TLR2 mutants, with β-actin as an internal control. (JPG) [file pone.0306096.s001.jpg]
